# Lab-Attenuated Rabies Virus Causes Abortive Infection and Induces Cytokine Expression in Astrocytes by Activating Mitochondrial Antiviral-Signaling Protein Signaling Pathway

**DOI:** 10.3389/fimmu.2017.02011

**Published:** 2018-01-19

**Authors:** Bin Tian, Ming Zhou, Yu Yang, Lan Yu, Zhaochen Luo, Dayong Tian, Ke Wang, Min Cui, Huanchun Chen, Zhen F. Fu, Ling Zhao

**Affiliations:** ^1^State Key Laboratory of Agricultural Microbiology, Huazhong Agricultural University, Wuhan, China; ^2^Key Laboratory of Preventive Veterinary Medicine of Hubei Province, Huazhong Agricultural University, Wuhan, China; ^3^College of Veterinary Medicine, Huazhong Agricultural University, Wuhan, China; ^4^Department of Pathology, University of Georgia, Athens, GA, United States

**Keywords:** rabies virus, astrocytes, double-strand RNA, cytokine expression, mitochondrial antiviral-signaling protein signaling pathway

## Abstract

Rabies is an ancient disease but remains endemic in most parts of the world and causes approximately 59,000 deaths annually. The mechanism through which the causative agent, rabies virus (RABV), evades the host immune response and infects the host central nervous system (CNS) has not been completely elucidated thus far. Our previous studies have shown that lab-attenuated, but not wild-type (wt), RABV activates the innate immune response in the mouse and dog models. In this present study, we demonstrate that lab-attenuated RABV causes abortive infection in astrocytes, the most abundant glial cells in the CNS. Furthermore, we found that lab-attenuated RABV produces more double-stranded RNA (dsRNA) than wt RABV, which is recognized by retinoic acid-inducible gene I (RIG-I) or melanoma differentiation-associated protein 5 (MDA5). Activation of mitochondrial antiviral-signaling protein (MAVS), the common adaptor molecule for RIG-I and MDA5, results in the production of type I interferon (IFN) and the expression of hundreds of IFN-stimulated genes, which suppress RABV replication and spread in astrocytes. Notably, lab-attenuated RABV replicates in a manner identical to that of wt RABV in MAVS−/− astrocytes. It was also found that lab-attenuated, but not wt, RABV induces the expression of inflammatory cytokines *via* the MAVS- p38/NF-κB signaling pathway. These inflammatory cytokines increase the blood–brain barrier permeability and thus enable immune cells and antibodies infiltrate the CNS parenchyma, resulting in RABV control and elimination. In contrast, wt RABV restricts dsRNA production and thus evades innate recognition by RIG-I/MDA5 in astrocytes, which could be one of the mechanisms by which wt RABV evades the host immune response in resident CNS cells. Our findings suggest that astrocytes play a critical role in limiting the replication of lab-attenuated RABV in the CNS.

## Introduction

Rabies is an acute encephalomyelitis. The hallmark of rabies is that the disease is almost always fatal once clinical signs develop (1, 2). The causative agent, rabies virus (RABV), is a negative-strand RNA virus belonging to the genus *Lyssavirus* in the family *Rhabdoviridae* (3). RABV enters neurons from the neuromuscular junction closest to the site of infection. After a short incubation, RABV travels to the central nervous system (CNS) through sensory or motor neurons. Only slight tissue damage and neuroinflammation can be observed in the brains of rabid patients (4). In contrast, lab-attenuated RABV induces extensive inflammation and apoptosis, as well as increases in the expression levels of innate immunity-related genes in the CNS of infected mice (5–10). These findings suggest that wt, but not lab-attenuated, RABV evades the host immune responses.

The innate immune system is the first line of defense against viral invasion. Viruses are usually confronted by various pattern recognition receptors (PRRs), including Toll-like receptors (TLRs) and retinoic acid-inducible gene I (RIG-I) like helicases (RLRs) (11). TLR family members, such as TLR3 and TLR7, are generally involved in recognizing negative-strand RNA viruses (12). TLR3 binds double-stranded RNA (dsRNA), whereas TLR7 recognizes single-strand RNA. The main sources of dsRNA in infections with single-strand RNA viruses are the replicative intermediates generated by viral RNA-dependent RNA polymerase (13). TLR3 and TLR7 initiate signaling though the adaptor molecules TRIF and MyD88, respectively. Both signaling cascades triggered by these proteins lead to IRF3 phosphorylation in the C terminal region at serine 386, which is critical for IRF3 activation by two IκB kinases (TBK-1 and IKKε) (14, 15). Activated IRF3 homo- or hetero-dimerizes with IRF7 and then translocates into the nucleus, to interacts with the CREB binding protein CBP/p300 and stimulates the transcription of interferon (IFN)-β, as well as some IFN-stimulated genes (ISGs) (15, 16). Alternatively, RNA viruses can be recognized by two RLRs, RIG-I, and melanoma differentiation-associated protein 5 (MDA5), located in the cytoplasm (17, 18). RIG-I solely senses short and blunt dsRNA of negative-strand RNA viruses containing 5′ triphosphate RNA in the panhandle region of their single-stranded genome (17). Unlike RIG-I, MDA5 preferentially binds blunt-ended dsRNA, with or without 5′ triphosphate (18). RIG-I and MDA5 signaling is mediated through mitochondrial antiviral-signaling protein (MAVS), which is also known as IPS1, VISA or Cardif (19). Similar to TLR signaling, RLR signaling results in IRF3 activation and nuclear translocation (20).

Several groups have attempted to identify the PRRs that recognize RABV. Prehaud et al. found that TLR3 mRNA expression is upregulated following RABV infection in post-mitotic human neurons (21). Furthermore, enhanced TLR3 expression has also been observed in the cerebellar cortical tissues of rabies patients (22). The observation that TLR3 is upregulated following infection suggests that TLR3 plays a role in the innate recognition of RABV. Faul et al. found that both RIG-I and MDA5 were responsible for inducing dendritic cell (DC) activation and type I IFN production upon RABV infection (23). MAVS, the adaptor protein for both RIG-I and MDA5, is essential for inducing innate immune responses in DCs. In another study, Li et al. found that mice lacking TLR7 exhibited a phenotype associated with intermediate mortality rates between those of MyD88−/− and control mice, indicating that TLR7 may play an important role in controlling RABV infections. However, the role of TLR7 in RABV infection is not entirely understood (24).

Astrocytes are the most abundant glial cells in the CNS and constitute the blood–brain barrier (BBB) along with endothelial cells and pericytes (25). Astrocytes are generally involved in regulating the CNS microenvironment and also play roles in neuronal metabolic support, synaptic transmission, and neurotropism. Moreover, astrocytes participate in developing and maintaining the BBB and guiding neuronal migration during development. The roles of astrocytes in innate immunity and inflammation have been reported recently (26). Astrocytes are the reservoir for many neuroinvasive viruses, such as human immunodeficiency virus, Theiler’s murine encephalomyelitis virus (TMEV), John Cunningham virus, and herpes simplex virus (HSV) (27–30). Astrocytes have also been shown to play multiple roles in viral infections. Specifically, they can increase BBB permeability (31) by producing cytokines and degrading tight junction proteins (32). Moreover, astrocytes have been shown to induce the innate immune response to produce IFN (33). An early report showed that both wt and lab-attenuated RABV could successfully infect primary astrocytes in the early stages of RABV infection (34). A recent study demonstrated that a RABV vaccine strain SAD-L16 caused an abortive infection in astrocytes, but the detailed mechanism was not revealed (35). In this study, it was found that lab-attenuated RABV produces higher level of dsRNA than wt RABV, which is recognized by RIG-I/MDA5 and results in the activation of MAVS signaling pathway. Following MAVS activation, the production of ISG and inflammatory cytokines helps to clear the lab-attenuated RABV from the CNS. In contrast, wt RABV can maintain a persistent infection in astrocytes by evading the innate recognition.

## Materials and Methods

### Cells, Viruses, Antibodies, and Mice

Mouse bEnd.3 cells and Vero cells were obtained from the American Type Culture Collection (ATCC; Manassas, VA, USA) and were maintained in Dulbecco’s modified Eagle medium (DMEM) supplemented with fetal bovine serum (FBS; Gibco, Carlsbad, CA, USA). Mouse neuroblastoma (NA) cells were maintained in RPMI-1640 medium (Thermo-Fisher, USA) supplemented with 10% FBS.

The DRV-AH08 (DRV) was isolated from a rabid dog in Anhui province, China (36, 37). CVS-B2c (B2c), originated from CVS-24 virus by passage in BHK-21 cells (6), has been used as a lab-attenuated RABV (38–40). Both DRV and CVS-B2c were propagated in suckling ICR mouse brains. All the viruses were manipulated under the standard biosecurity procedures made by The Ministry of Agriculture of China.

A rabbit anti-ubiquitin protein C (UBC) polyconal antibody was purchased from ABclonal Technology (Woburn, MA, USA). A rabbit anti-RIG-I monoclonal antibody was purchased from Enzo Life Technology (Farmingdale, NY, USA), and rabbit polyclonal antibodies against IRF7, STAT1 and Occludin were obtained from Santa Cruz Biotechnology (Santa Cruz, CA, USA). A rabbit antiphospho-IRF7 monoclonal antibody was purchased from Cell Signaling Technology (Washington, DC, USA), and rabbit anti-IFIT1 and IRF3 monoclonal antibodies were purchased from Abcam (Cambridge, MA, USA). A rabbit polyclonal anti-claudin-5 antibody, a biotinylated goat anti-rabbit or mouse 594 antibody, and an Alexa Fluor 488-conjugated goat antimouse or rabbit antibody were purchased from Invitrogen (Grand Island, NY, USA). Mouse monoclonal anti-Zoluna occludens-1 (anti-ZO-1) antibodies were obtained from Sigma (St. Louis, MO, USA), and mouse monoclonal anti-GAPDH antibody was purchased from ProteinTech (Wuhan, China). The mouse anti-RABV N and P monoclonal antibodies were prepared in our laboratory, and the fluorescein isothiocyanate (FITC)-conjugated anti-RABV nucleoprotein antibody used herein was obtained from Fujirebio (Malvern, PA, USA).

Female C57BL/6 mice (6–8 weeks old) were purchased from the Center for Disease Control of Hubei province (Wuhan, China), and MAVS-knockout (MAVS−/−, Stock No: 008634-B6; 129-*Mavs^tm1Zjc^*/J, USA) and TLR7-knockout (TLR7−/−, Stock No: 008380-B6; 129S1-*Tlr7^tm1Flv^*/J, USA) mice were purchased from the Jackson Laboratory (Bar Harbor, ME, USA). All the mice were housed in the animal facility at Huazhong Agricultural University, Wuhan, China.

### Mouse Infections

Female wt or MAVS-knockout (MAVS−/−) C57BL/6 mice (6–8 weeks old) were infected intracerebally (i.c.) with 20 µL of DRV-AH08 (200 FFU), B2c (20 FFU), or mock infected with the same volume of DMEM. At 7 days postinfection (d.p.i.), the mice were euthanized with CO_2_ when moribund, and their brains were collected for immunohistochemistry analysis.

### BBB Permeability Assay

Blood–brain barrier permeability was determined by measuring sodium fluorescein (NaF) uptake as described previously with minor modifications (41). Briefly, 100 µl of NaF (100 mg/ml) was injected intraperitoneally (i.p.) into each mouse. After anesthetization, peripheral blood and brains of each mouse were collected. The fluorescence in serum and brain homogenate samples was analyzed by a spectrophotometer (BioTek Instruments, VT, USA) with excitation at 485 nm and emission at 530 nm. Standards (125–4,000 g/ml) were prepared to calculate the NaF content. NaF uptake into tissue is calculated as (μg of fluorescence spinal cord/mg of tissue)/(μg of fluorescence sera/ml of blood) to normalize values for blood levels of the dye at the time of tissue collection.

### Immunohistochemistry

To detect CD45^+^ cell in the brain, infected mice were anesthetized with ketamine-xylazine (0.1 ml/10 g body weight), perfused with 50 ml PBS, and then the brains were transferred into 4% neutral buffered paraformaldehyde (PFA) for at least 24 h (39). Briefly, the brain sections were antigenic recovered and blocked with donkey serum, incubated with primary antibodies overnight at 4°C, and then secondary antibodies was applied. PBS was treated as a negative control by replacing primary antibodies. Sections were photographed and analyzed using an Olympus BX41 microscope (Tokyo, Japan).

### Primary Astrocyte Isolation

Primary mixed glial cell cultures were established as described previously (42). Briefly, the brain cells of 1- to 3-day-old neonatal C57BL/6 mice were dissociated by repeated pipetting and then passed through a 75-nm nylon mesh (Corning, NY, USA). The cells were subsequently washed once in cold PBS and cultured in DMEM (with high glucose) supplemented with 10% FBS and 1% penicillin–streptomycin. The medium was changed on days 3, 5, and 7 for the astrocytes and on day 3 only for the microglia. On day 10, the flasks were shaken at 260 rpm for 2 h to remove any non-adherent cells (mainly microglia). The remaining adherent astrocytes were detached with trypsin-EDTA and then plated again for further experiments. The purity of the astrocyte cultures was greater than 95%.

### Neuron Isolation

Mouse neurons were obtained from embryonic mouse brains as previously described (42) and then dissociated by repeated pipetting (approximately 20 times) before being passed through a 75-nm nylon mesh. The cells were then washed once in cold PBS and cultured in DMEM (with high glucose) supplemented with 5% FBS and 1% penicillin–streptomycin for 6 h, after which the medium was replaced with serum-free neural-basal medium (Invitrogen, Carlsbad, CA, USA) supplemented with 2% B-27 (Invitrogen, Carlsbad, CA, USA).

### Virus Titration

Viral titers were determined by direct fluorescent antibody assay (40). NA cells cultured in 96-well plates were inoculated with viruses diluted serial 10-fold and then incubated at 37°C for 48 h. Then the culture supernatant was subsequently removed, and the cells were fixed and stained with FITC-conjugated anti-RABV N antibodies. The antigen-positive foci were counted under a fluorescence microscope (Zeiss, Germany), and the viral titers were calculated as FFU per milliliter. All titrations were conducted in quadruplicate.

### Confocal Microscopy

Primary astrocytes, neurons or bEnd.3 cells were seeded on coverslips and allowed to grow until they formed a confluent monolayer, after which they were infected with RABV or suspended in medium containing the supernatants of infected astrocytes. The cells were subsequently fixed with 4% PFA, permeabilized with 0.1% Triton X-100 and then stained with antibodies against dsRNA, RABV N, RABV P, Claudin-5, Occludin, ZO-1, or DAPI.

Infected wt or MAVS−/− mice were anesthetized with ketamine-xylazine (0.1 mL/10 g body weight) and then perfused with PBS followed by 10% neutral buffered formalin, as described previously (39). Three independent mouse brain samples were collected from each group and embedded in paraffin for coronal sectioning. The sections were subsequently stained with antibodies against GFAP, MAP2, RABV P, RABV N, or DAPI. After being washed, the cells or sections were incubated with an Alexa Fluor 488-conjugated goat antirabbit or mouse secondary antibodies or an Alexa Fluor 594-conjugated goat antirabbit or mouse secondary antibodies for 1 h at room temperature. Staining was visualized with a Nikon A1 confocal laser microscope system equipped with NIS-Elements imaging software (Nikon, Melville, NY, USA) and was quantified using Fiji, an ImageJ distribution package manufactured by NIH (http://imagej.net/Introduction). Mean fluorescence intensity (MFI) was quantified using the region of interest, which encompassed the entire cell to include the membrane, and background staining was quantified using three negatively stained regions per cell. These regions were subtracted from the total MFI.

### Inhibitor Treatment

For the protein synthesis inhibition tests, primary astrocytes were pretreated with cycloheximide (CHX) (Invivogen, San Diego, CA, USA) for 1 h at a dose of 50 µg/mL, infected with DRV or B2c at an MOI of 0.1 and then continued to be incubated with CHX for another 24 h. For the TBK1 activation blockage assays, BX795 (Invivogen, San Diego, CA, USA) at a dose of 1 µM was used to treat primary astrocytes.

For the inflammatory pathway blockage assays, the primary astrocytes were pretreated with a p38 inhibitor (Skepinone-L; SELLEK, Houston, TX, USA), a JNK inhibitor (JNK inhibitor IX; SELLEK, Houston, TX, USA), an NF-κB inhibitor (sc75741; SELLEK, Houston, TX, USA), or DMSO as control at a dose of 5 µg/mL for 1 h. Then the cells were infected with DRV or B2c at MOI 1 and incubated with the above inhibitors for 48 h.

### ELISA

The concentrations of TNF-α and IL-6 in the supernatant of astrocytes were measured by the commercial ELISA kits according to the manufacturer’s instruction (ABclonal Technology, Woburn, MA, USA).

### qRT-PCR

RNA was isolated with TRIzol^®^ Reagent (Invitrogen), according to the manufacturer’s instructions, and qRT-PCR was performed as described previously (39). Briefly, 800 ng of total RNA (from either cells or tissue) was transcribed into cDNA in a reaction mixture with a total of volume of 20 µL using a Superscript III Reverse Transcription Kit (Toyobo). The reaction mixture comprised 1 μL of cDNA combined with 5 µL of iQ5 SYBR Green Mix (BioRad, Hercules, CA, USA), 3 µL of diethyl pyrocarbonate-treated water, and 0.5 µL of primer mix (the concentration of each primer was 10 mM). The cDNA was amplified using an iQ5 iCycler (Bio-Rad), and the cycle threshold (*CT*) values were recorded. The *CT* value was inversely correlated with the mRNA concentration, and each *CT* unit represented a twofold change in the mRNA concentration. Basal mRNA expression levels were expressed as ^Δ^*CT* values and were normalized to β-actin mRNA expression levels [^Δ^*CT CT* (ISG)/^Δ^*CT* (β-actin)]. Induced mRNA expression levels were expressed as fold changes relative to mock-infection levels using the 2^ΔΔ^*^CT^* method. All the primer sequences are listed in (Table 1). To quantify cellular RABV N RNA levels, we transcribed the total RNA using Avian myeloblastosis virus Reverse Transcriptase XL (TAKARA, Kusatsu, Japan) and a primer specific for the RABV N genomic sequence. A standard curve was generated from serially diluted plasmids carrying a RABV N gene and the copy numbers of N mRNA were normalized to 1 mg of total RNA.

**Table 1:** Primers used for quantification of viral mRNA, IFN-stimulated genes, chemokines, and cytokines.

Primer name	Sequence (5′–3′)	Use
Retinoic acid-inducible gene I (RIG-I) F	GCGTCTCAGTGCAGCACATCATT	qRT-PCR
RIG-I R	GGGTCCCGTGACTCTCCAAGTTT	qRT-PCR
Melanoma differentiation-associated protein 5 (MDA5) F	GCTGCTAAAGACGGAAATCG	qRT-PCR
MDA5 R	CTTGTCGCTGTCATTGAGGA	qRT-PCR
Mitochondrial antiviral-signaling protein (MAVS) F	ATGCCGTTTGCTGAAGAC	qRT-PCR
MAVS R	CTAGTGCAGACGCCGCCG	qRT-PCR
IRF7 F	CTGGAGCCATGGGTATGCA	qRT-PCR
IRF7 R	AAGCACAAGCCGAGACTGCT	qRT-PCR
Interferon (IFN)-β F	CACAGCCCTCTCCATCAAC	qRT-PCR
IFN-β R	GCATCTTCTCCGTCATCTCC	qRT-PCR
STAT1 F	GTGGACATTGAGTTCTTGGTGAGATCC	qRT-PCR
STAT1 R	CCTTTCTCATTCTGTCGACTTTGTTGG	qRT-PCR
IFIT1 F	GCATCACCTTCCTCTGGCTA	qRT-PCR
IFIT1 R	TGTTGTTCAGTGCCTTCTGG	qRT-PCR
Rabies virus (RABV) NF	ACACCGGCAACTACAAGACA	qRT-PCR and cDNA synthesis
RABV NR	ATGGTACTCCAGTTGGCACA	qRT-PCR
β-actin F	AGGTGACAGCATTGCTTCTG	qRT-PCR
β-actin R	GCTGCCTCAACACCTCAAC	qRT-PCR

### Western Blot

The cells were lysed with RIPA buffer containing protease inhibitors (Roche), and the protein concentrations were measured using a DC protein assay kit (Bio-Rad). Equal quantities of protein were resolved by 12 or 15% SDS-PAGE and then transferred to polyvinylidene difluoride membranes (Bio-Rad), which were blocked with 5% nonfat milk before being incubated with primary antibodies against RIG-I, phosphorylated IRF7 (p-IRF7), STAT1, IFIT1, RABV N, Claudin-5, Occludin, ZO-1, or GAPDH and then probed with the appropriate secondary antibodies. The blots were then visualized using ECL reagent (GE, Pittsburgh, PA, USA) and detected under an Intelligent dark box II (GE, Pittsburgh, PA, USA).

### Cytokine and Chemokine Protein Quantification

Primary astrocytes were infected with DRV or B2c at an MOI of 1 or mock infected, and the cell supernatants were collected at 24, 48, or 72 h p.i. The levels of several inflammatory cytokines, including TNF-α, IL-6, IL-1β, IL-17, IFN-γ, and vascular endothelial growth factor (VEGF), were quantified in the mock- or RABV-infected cell supernatants by a Quantibody Mouse Cytokine Array 1 Kit (RayBiotech, Norcross, GA, USA), according to the manufacturer’s protocol. The array was scanned using a GenePix 4000B (Molecular Devices, Axon Instruments, Silicon Valley, CA, USA), and the data were collected at several PMT values ranging from 540 to 790 gain using GenePix Pro-software. The 590-gain PMT scan generated the optimal standard curve, and the results of this scan were analyzed using Q-Analyzer Software for QAM-CYT-1 (RayBiotech).

### Transendothelial Permeability Assay

Transendothelial permeability assay was performed according to the methods described previously (43), with some modifications. B.END3 cells were grown on 3-μm-pore transwell filter inserts until they reached 100% confluency. The medium was then treated with UV-inactivated cell culture supernatants collected from RABV-infected astrocytes. After the cells had incubated for 48 h, they were treated apically with Dextran-FITC at a dose of 0.1 µg/mL for 30 min. The samples were then removed from the lower chamber and subjected to fluorescence measurement, which were performed using a fluorimeter (BioTek, Winooski, VT, USA; the excitation wavelength was 492 nm, and the emission wavelength was 520 nm). The fluorescence values for the experimental cells were subsequently compared to corresponding values for a control cell monolayer.

### Statistical Analysis

Data are expressed as the mean and standard error of the mean (SEM), and the significance of the differences between groups was evaluated by Student’s *t* test or one-way analysis of variance followed by Tukey’s *post hoc* test. The survival ratios were analyzed by Log-rank (Mantel-Cox) test. The asterisks indicated statistical significance (*, *P* < 0.05; **, *P* < 0.01; ***, *P* < 0.001). Graphs were plotted and analyzed using GraphPad Prism software, version 6.0 (GraphPad Software, La Jolla, CA, USA).

## Results

### Comparison of the Pathogenicity of DRV-AH08 and CVS-B2c in Mice

**Figure 1:**
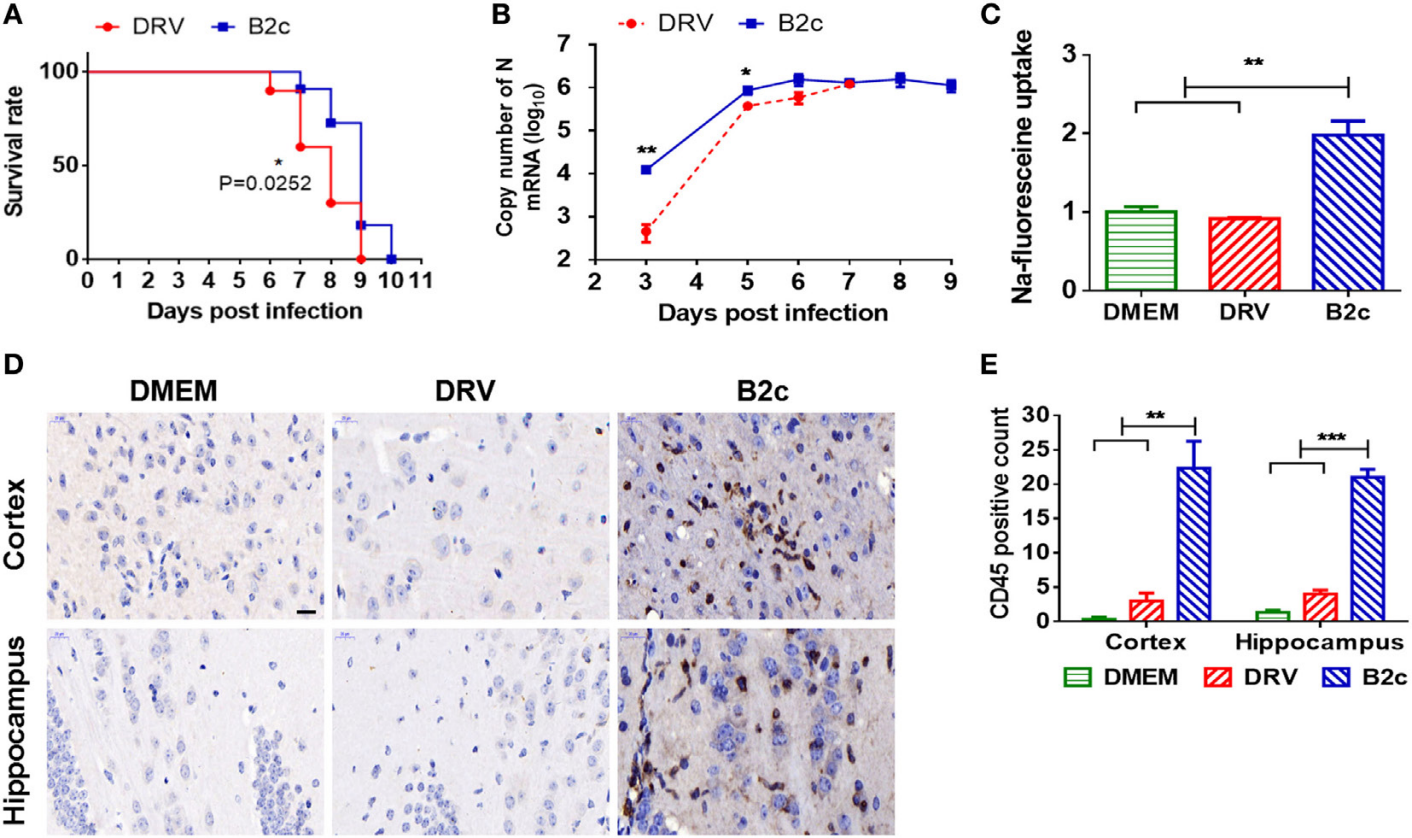
Wt and lab-attenuated rabies virus infection in mice. Groups of C57BL/6 mice (*n* = 10) were intracerebally (i.c.) inoculated with 20 FFU DRV or B2c, and observed daily for 2 weeks. **(A)** The survival ratio was presented and analyzed by Log-rank (Mantel-Cox) test. **(B)** Groups of C57BL/6 mice (*n* = 5) were i.c. infected with 200 FFU DRV or 20 FFU B2c. At indicated time points p.i., N mRNA of DRV and B2c in the mouse brain was analyzed by qRT-PCR. **(C)** The change of blood–brain barrier permeability in the DRV-, B2c-, and mock-infected mouse brain was determined by measuring the Na-fluorescence uptake. **(D)** The inflammation in DRV-, B2c-, and mock-infected mouse brains was analyzed by immunohistochemistry using anti-CD45 antibody. The scale bars represent 20 µm. **(E)** The inflammation was quantified by counting CD45^+^ lymphocytes from at least three different randomly selected areas.

First, the pathogenicity of the wt RABV strain DRV-AH08 (DRV) and lab-attenuated strain CVS-B2c (B2c) used in this study were compared in a mouse model. C57/BL6 mice were i.c. inoculated with 20 FFU B2c or DRV and the development of rabies was observed. As expected, DRV-infected mice displayed development of the diseases at 5 d.p.i. and all moribund at 9 d.p.i., earlier than B2c-infected mice which all succumb to rabies at 10 d.p.i. (Figure 1A).

We found that B2c replicated faster than DRV at the early stage of the infection in the CNS. To ensure that the viral load in the brains is similar, C57/BL6 mice were i.c. inoculated with 20 FFU B2c or 200 FFU DRV. Then the viral load in the mice brain were determined at 3, 5, 6, 7, 8, and 9 d.p.i. The results showed that the genomic RNA of DRV was lower than that of B2c at 3 and 5 d.p.i., but reached the same level as that of B2c at 6 and 7 d.p.i. (Figure 1B). Previous studies have shown that the lab-attenuated RABV infection enhances the BBB permeability and induces inflammation in the brain (8, 38). Thus, the Na-fluorescence (Na-F) uptake of both strains at 7 d.p.i. was measured, and the data showed that Na-F uptake in B2c-infected brain was significantly higher than that of DRV or mock-infected brains (Figure 1C). Consistently, there were more CD45^+^ lymphocytes in B2c-infected brains than DRV-infected mouse brains (Figures 1D,E). All these results are consistent with the previous findings related to the CNS inflammation and BBB permeability change during comparison of the pathogenicity between wt and lab-attenuated RABV (6, 8, 39).

### B2c Causes Abortive Infection, but DRV Can Persistently Replicate in Primary Astrocytes

**Figure 2:**
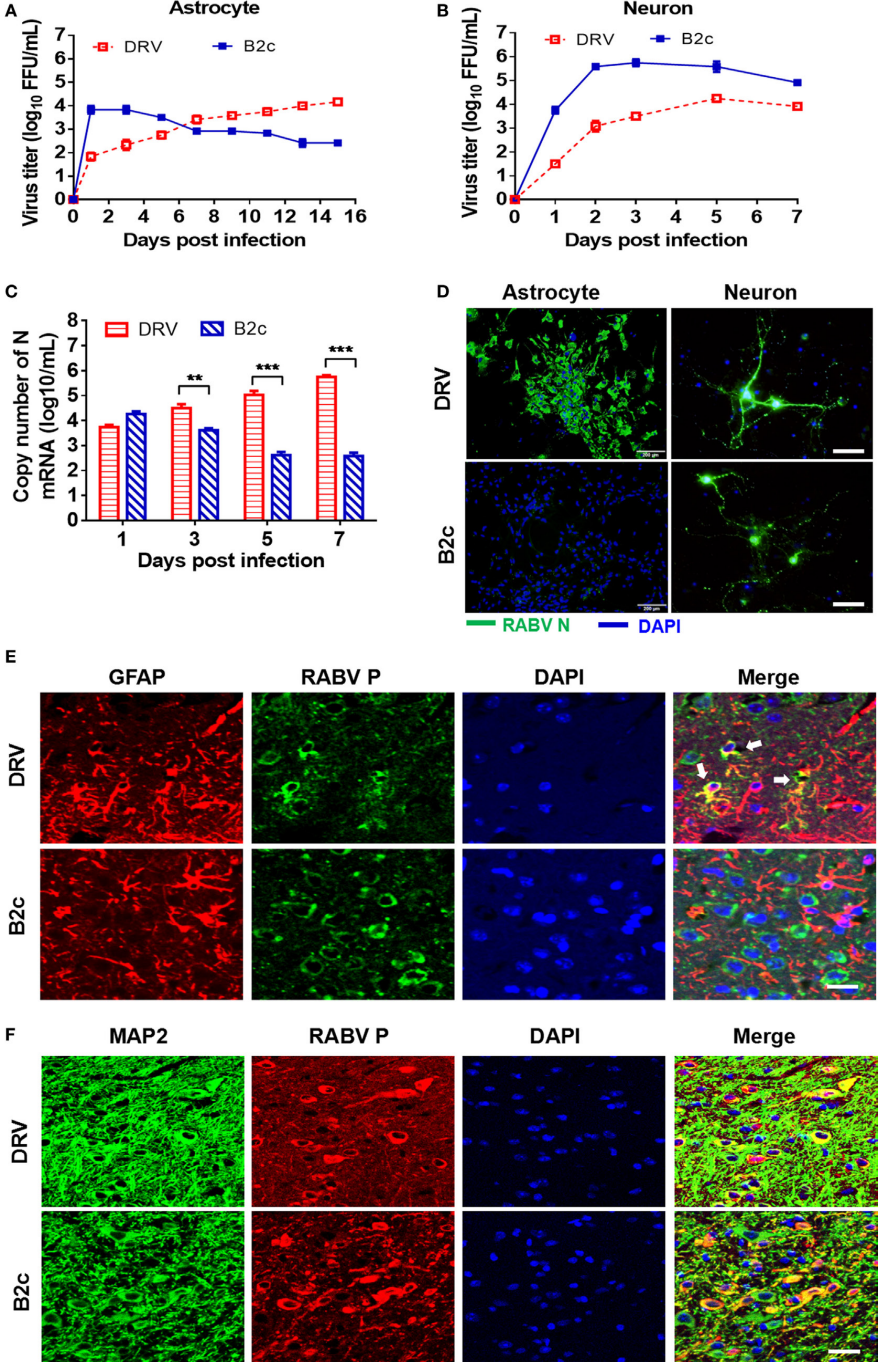
Lab-attenuated rabies virus (RABV) causes abortive infection in astrocytes. Primary astrocytes and neurons were isolated from suckling C57BL/6 mice and then infected with DRV or B2c at an MOI of 0.01. **(A,B)** The cell supernatants from virus infected astrocytes and neurons were collected at indicated time points, and viral titers were determined. **(C)** RABV N transcription levels in astrocytes were determined by qRT-PCR. **(D)** At the 7 days postinfection (d.p.i.), astrocytes were fixed with 4% PFA, stained with an FITC-conjugated anti-RABV N protein antibody (N-FITC) or DAPI and then observed under a fluorescence microscope. The scale bars represent 200 µm. At 24 h p.i., neurons were fixed with 4% paraformaldehyde (PFA) and stained with N-FITC or DAPI. The scale bars represent 50 µm. Female C57BL/6 mice were infected with DRV (200 FFU) or B2c (20 FFU). **(E)** At 7 d.p.i., mice were euthanized, perfused with PBS and then fixed with 4% PFA. The brains were subsequently coated with paraffin, and the brain sections were stained with antibodies against GFAP (red), RABV P protein (green), or DAPI (blue). The white arrows indicate RABV-infected astrocytes. The scale bars represent 50 µm. **(F)** The same brain sections as **(E)** were stained with antibodies against MAP2 (green), RABV P protein (red), or DAPI (blue), then visualized under a confocal microscope. The scale bars represent 50 µm.

Astrocytes play an important role in the induction of innate immunity in the CNS. To investigate the role of astrocytes in the pathogenesis of RABV, we isolated primary astrocytes and neurons from suckling mice and infected them with DRV or B2c. The growth kinetics of both viruses in astrocytes was assessed by virus titration and immunofluorescence assay (IFA). The viral loads in the cell culture supernatants infected with B2c quickly reached peak at 1 d.p.i., and then gradually decreased until 15 d.p.i. In contrast, the virus titer of DRV was initially relatively low but subsequently increased steadily until the endpoint (Figure 2A). To be noted, the viral titer of DRV in the cell supernatant maintained at a relatively low level than that of B2c in astrocyte at 3 and 5 d.p.i., but N mRNA transcription levels of DRV was significantly higher than that of B2c at 3 and 5 d.p.i. (Figure 2C). The IFA results showed that DRV persistently replicated in astrocytes and large immunofluorescence foci could be observed at 7 d.p.i., while no obvious immunofluorescence foci could be found in B2c-infected cells (Figure 2D). As a control, virus replication kinetics in primary neurons was also compared. It was found that viral titers of B2c were always higher than those of wt RABV at the indicated time points (Figure 2B). The IFA results also showed that both DRV and B2c could efficiently replicate in neuron (Figure 2D).

To confirm these observations *in vivo*, C57BL/6 mice were i.c. infected with 20 FFU B2c or 200 FFU DRV. Infected mice were euthanized when moribund and the brains were harvested for fluorescence IHC analysis. GFAP was a well-known surface marker for astrocytes (44), and the GFAP staining demonstrated DRV could effectively infect astrocytes in the brains (Figure 2E). However, few infected astrocytes could be observed in B2c-infected mouse brains (Figure 2E). In contrast, neuron was intensively infected by both DRV and B2c (Figure 2F). Together, these results show that the B2c causes abortive infection in astrocytes both *in vitro* and *vivo*.

### Lab-Attenuated RABV Activates the MAVS Signaling Pathway in Astrocytes

**Figure 3:**
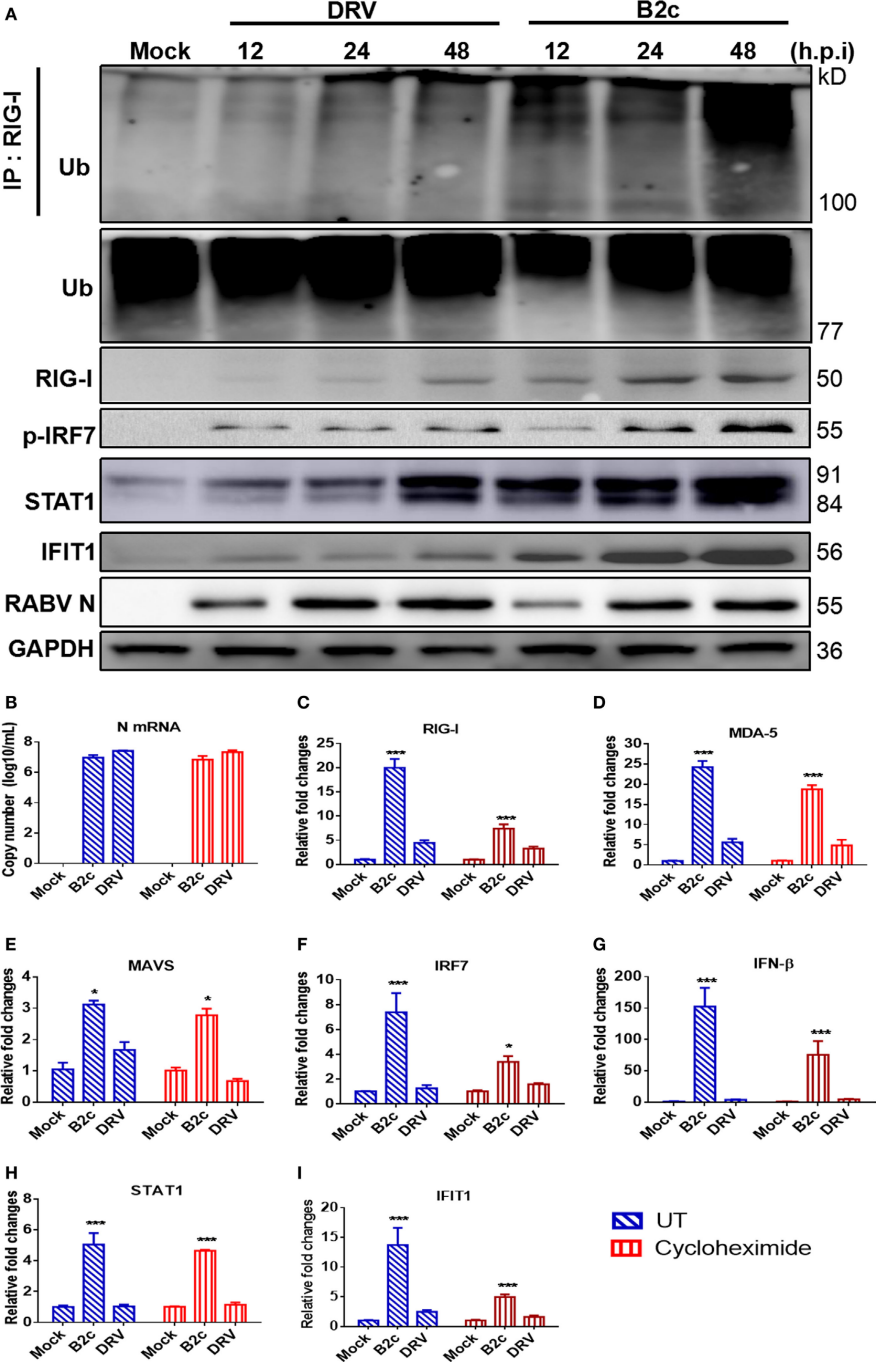
Lab-attenuated rabies virus (RABV) causes the activation of mitochondrial antiviral-signaling protein (MAVS) signaling pathway in astrocytes. Primary astrocytes were infected with DRV or B2c at an MOI of 0.1. **(A)** At the indicated time points, the cells were lysed with RIPA buffer, the activation of retinoic acid-inducible gene I (RIG-I) was characterized by immunoprecipitation with an anti-RIG-I antibody and then immunostaining with an anti-UBC antibody. The protein expression level of the key molecules in MAVS signaling pathway was assessed by Western blot using antibodies against RIG-I, phosho-IRF7, STAT1, IFIT1, RABV N, and GAPDH. **(B)** RABV N mRNA transcription levels were measured by qRT-PCR after the cells were treated with cycloheximide or mock-controls. **(C–I)** The fold changes in RIG-I, melanoma differentiation-associated protein 5, MAVS, IRF7, interferon-β, STAT1, and IFIT1 transcription levels were measured by qRT-PCR. The qRT-PCR results were calculated by the ΔΔC_t_ method and expressed as 2^−ΔΔCt^.

Previous studies have demonstrated that RABV can be recognized by RIG-I and MDA5, which share a common adaptor MAVS in DCs (23). To assess innate immune responses in astrocytes, cells were infected with DRV or B2c at an MOI of 0.1 and the expression of several proteins involved in the MAVS signaling pathway, namely, RIG-I, p-IRF7, STAT1 and IFIT1 (ISG56), was measured by Western blot. The ubiquitin ligase TRIM25 mediates Lysine 63-linked ubiquitination of RIG-I’s N-terminal CARD domains is indispensable to induce type I IFN production and antiviral immunity (45). Thus, the immunoprecipitation of RIG-I was carried out and then resolved by Western blot by using an anti-ubiquitin antibody. As expected, RIG-I was much more robustly ubiquitinated in astrocytes infected with B2c than that in astrocytes infected with DRV. Consistently, the expression levels of RIG-I, p-IRF7, STAT1, and IFIT1 in B2c-infected astrocytes were higher than those in DRV-infected cells (Figure 3A).

Next, we attempted to determine which viral product (RNA or protein) activates MAVS signaling pathway in astrocytes. Primary astrocytes were treated with CHX, a protein synthesis inhibitor, to inhibit viral protein synthesis. It was found that B2c and DRV N transcription levels were similar between CHX-treated and mock-treated astrocytes at 24 h p.i. (Figure 3B). However, the expression levels of the genes involved in the MAVS signaling pathway, namely, RIG-I, MDA5, MAVS, IRF7, IFN-β, STAT1, and IFIT1, were significantly upregulated by B2c compared with DRV in both CHX-treated and mock-treated astrocytes, indicating that viral RNA rather than proteins activates IFN pathway depending on MAVS (Figures 3C–I). Taken together, these data demonstrate that the lab-attenuated RABV, but not wt RABV, activates the MAVS signaling pathway by viral RNA, resulting in the production of IFN as well as ISGs in astrocytes.

### Lab-Attenuated RABV Produces More dsRNA in Astrocytes

**Figure 4:**
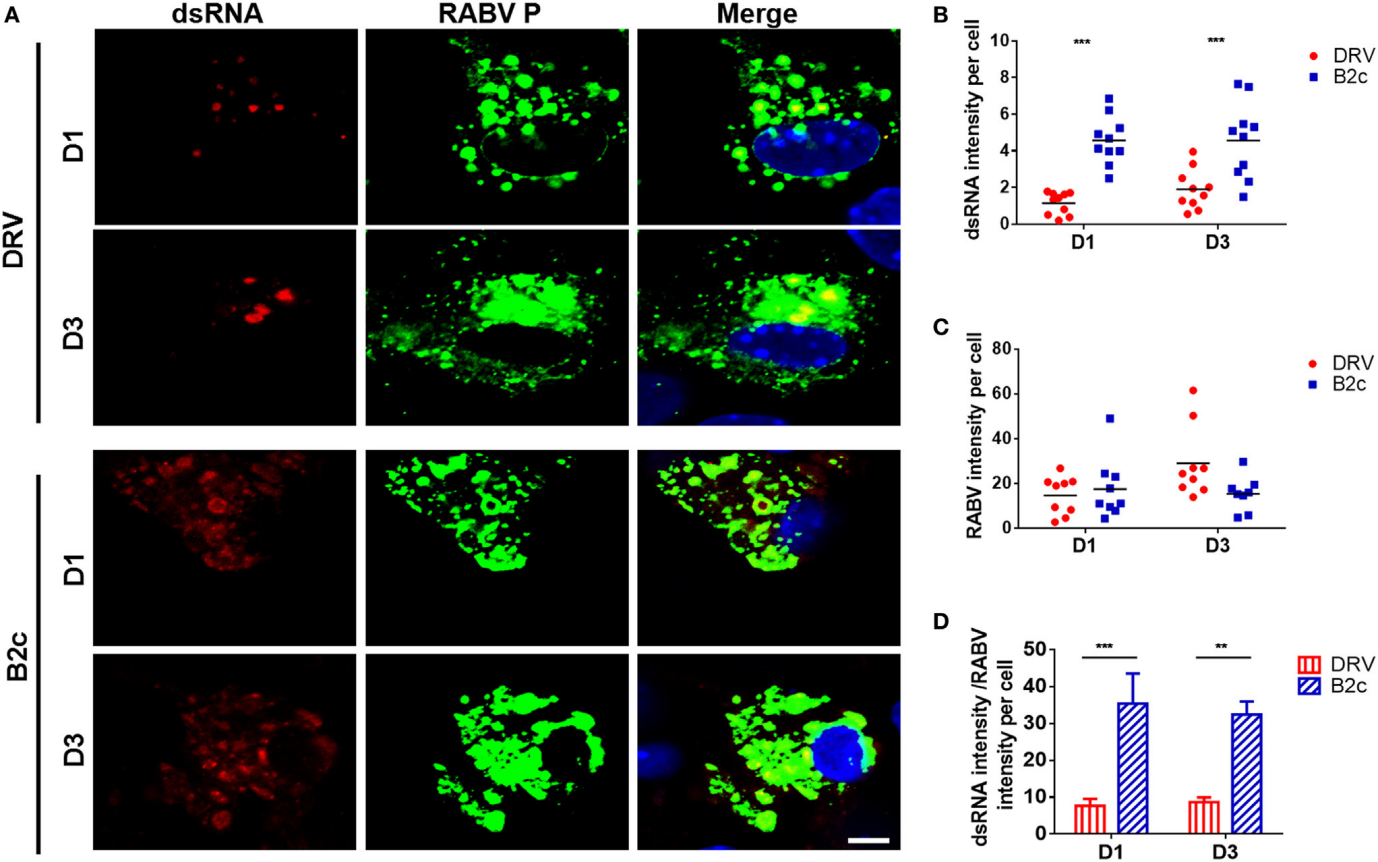
Lab-attenuated rabies virus (RABV) induces double-stranded RNA (dsRNA) production in astrocytes. Primary astrocytes were infected with DRV or B2c at an MOI of 0.1. **(A)** At 1 and 3 days postinfection (d.p.i.), the cells were fixed with 4% paraformaldehyde and stained with antibodies against dsRNA, RABV P, or DAPI, then visualized under a confocal microscope. The scale bars represent 10 µm. **(B,C)** The fluorescence intensity of dsRNA or RABV was normalized and analyzed from ten different individual astrocytes by using Image J. **(D)** Ratios of the dsRNA intensity to the RABV intensity per cell derived from **(B,C)** was calculated.

Retinoic acid-inducible gene I and MDA5 activation is induced mostly by dsRNA, which is produced during viral replication (13). To compare the amount of dsRNA produced by wt and lab-attenuated RABV, primary astrocytes were infected with DRV or B2c at an MOI of 0.1. At 1 and 3 d.p.i., dsRNA was stained with specific antibody and observed by a confocal fluorescence microscope. The results demonstrate that more dsRNA is synthesized in B2c-infected astrocytes than those in DRV-infected cells (Figure 4A). The dsRNA intensity per cell in B2c-infected cell was significantly higher than that in DRV-infected cells (Figure 4B), considering the similar RABV intensity per cell (Figure 4C). The ratio of dsRNA intensity to RABV intensity in B2c-infected astrocyte was significantly higher than that in DRV-infected cells (Figure 4D). These results suggest that lab-attenuated RABV produces more viral dsRNA than wt RABV during viral replication, which causes the activation of MAVS as well as the downstream signaling.

### Lab-Attenuated RABV Can Effectively Replicate in MAVS Knock-Out Astrocytes

**Figure 5:**
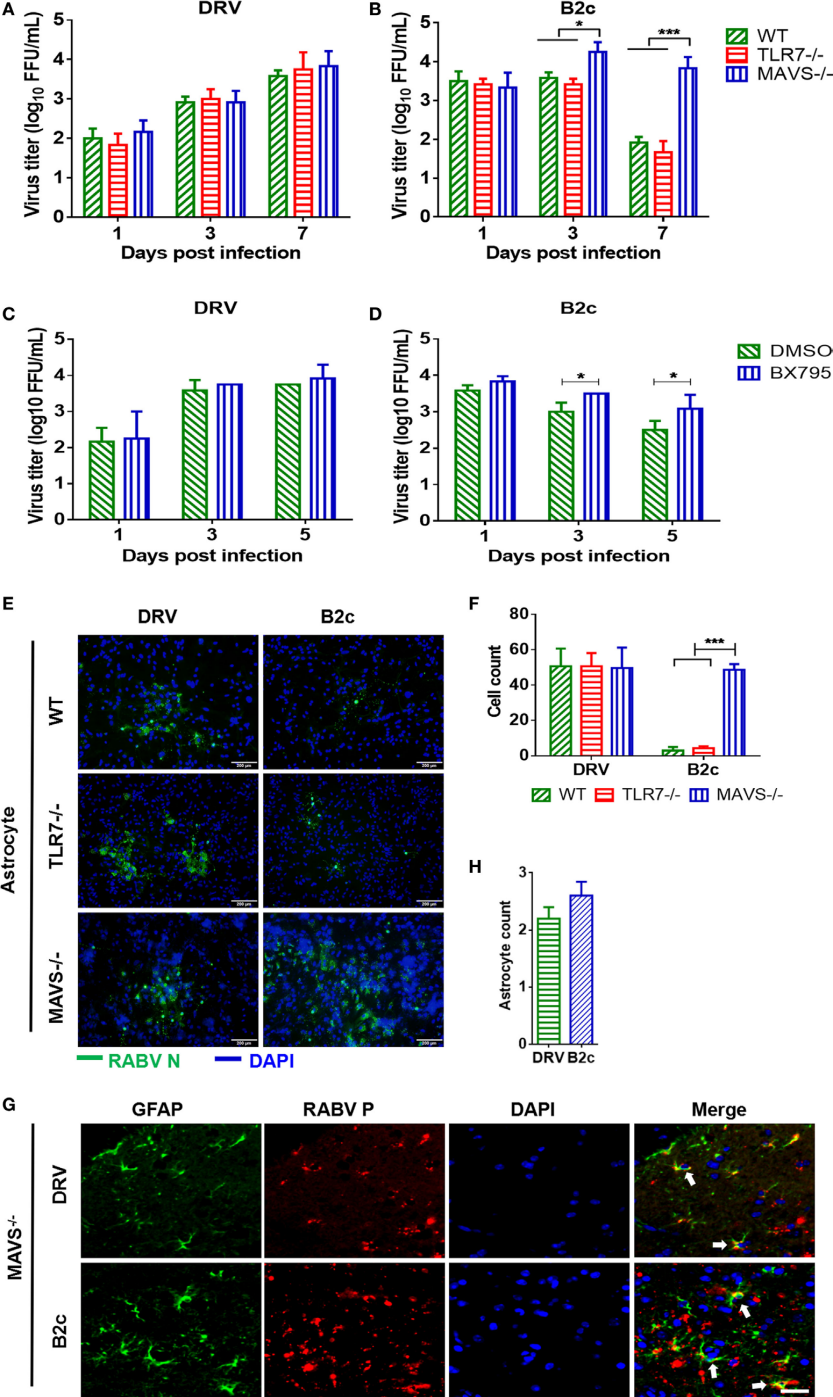
Lab-attenuated rabies virus (RABV) infects astrocytes lacking mitochondrial antiviral-signaling protein (MAVS) in the same manner as wt RABV. **(A,B)** Wild-type (wt), MAVS−/−, or TLR7−/− astrocytes were infected with DRV or B2c at an MOI of 0.01. At the indicated time points, the viral titers in the cell culture supernatant were determined by virus titration. **(C,D)** Primary astrocytes were treated with BX795 at a dose of 1 µM or DMSO, and then infected with DRV or B2c at an MOI of 0.01, at the indicated time points, the viral titers in the cell culture supernatant were determined by virus titration. **(E,F)** Wt, TLR7−/− or MAVS−/− astrocytes were infected with DRV or B2c at an MOI of 0.01, fixed with paraformaldehyde and then stained with anti-RABV N antibodies or DAPI, and the viral infected cells were calculated from five different areas. The scale bars represent 200 µm. **(G,H)** At 7 days postinfection, the cerebral cortex sections from MAVS−/− mice were subsequently stained with antibodies against GAFP (red), RABV P (green), or DAPI (blue), and then visualized under a confocal microscope, and the viral infected astrocytes were calculated from five different areas. The white arrow indicates RABV-infected astrocytes. The scale bars represent 50 µm. The infected cells were quantified by ImageJ software and statistical analysis was applied.

To evaluate the role of MAVS signaling on RABV infections in astrocytes, primary astrocytes isolated from MAVS−/− and wt mice were infected with DRV or B2c at an MOI of 0.01. At 1, 3, and 7 d.p.i., viral titers in the supernatant were measured. Notably, B2c titers were significantly increased in MAVS−/− astrocytes compared with wt astrocytes (Figures 5A,B). Moreover, the cell numbers of immunofluorescence plaques in MAVS−/− astrocytes caused by B2c infection were significantly more than those in wt astrocytes (Figures 5E,F). In contrast, MAVS deficiency had no significant influence on DRV replication and spread in astrocytes (Figures 5A,E). TBK1 is an IκB kinase downstream of the MAVS signaling pathway and is critical for IRF3 phosphorylation. Treatment with the TBK1 specific inhibitor BX795 significantly increased B2c titers in astrocytes (Figures 5C,D). To verify these observations *in vivo*, MAVS−/− mice were i.c. infected with DRV or B2c, and virus infection of astrocytes was determined by IFA. We found that both DRV and B2c could efficiently infected MAVS−/− astrocytes (Figures 5G,H). Taken together, these findings suggest that MAVS signaling significantly restricts the replication and spread of lab-attenuated but not wt RABV in astrocytes.

Recent studies have shown that TLR7 may be another innate immune molecule that recognizes RABV (24). Thus, the role of TLR7 in RABV replication was investigated in astrocytes. Astrocytes from TLR7−/− and control mice were isolated and then infected with DRV or B2c at an MOI of 0.01. At different time points p.i., viral titers (Figures 5A,B) and the formation of viral immunofluorescence foci was analyzed (Figures 5E,F). The results demonstrated that TLR7 deficiency did not significantly affect the replication and spread of either DRV or B2c in astrocytes, suggesting that TLR7 does not play a role in containing RABV replication and spread in astrocytes.

### Lab-Attenuated RABV Induces Cytokine Production in Astrocytes

**Figure 6:**
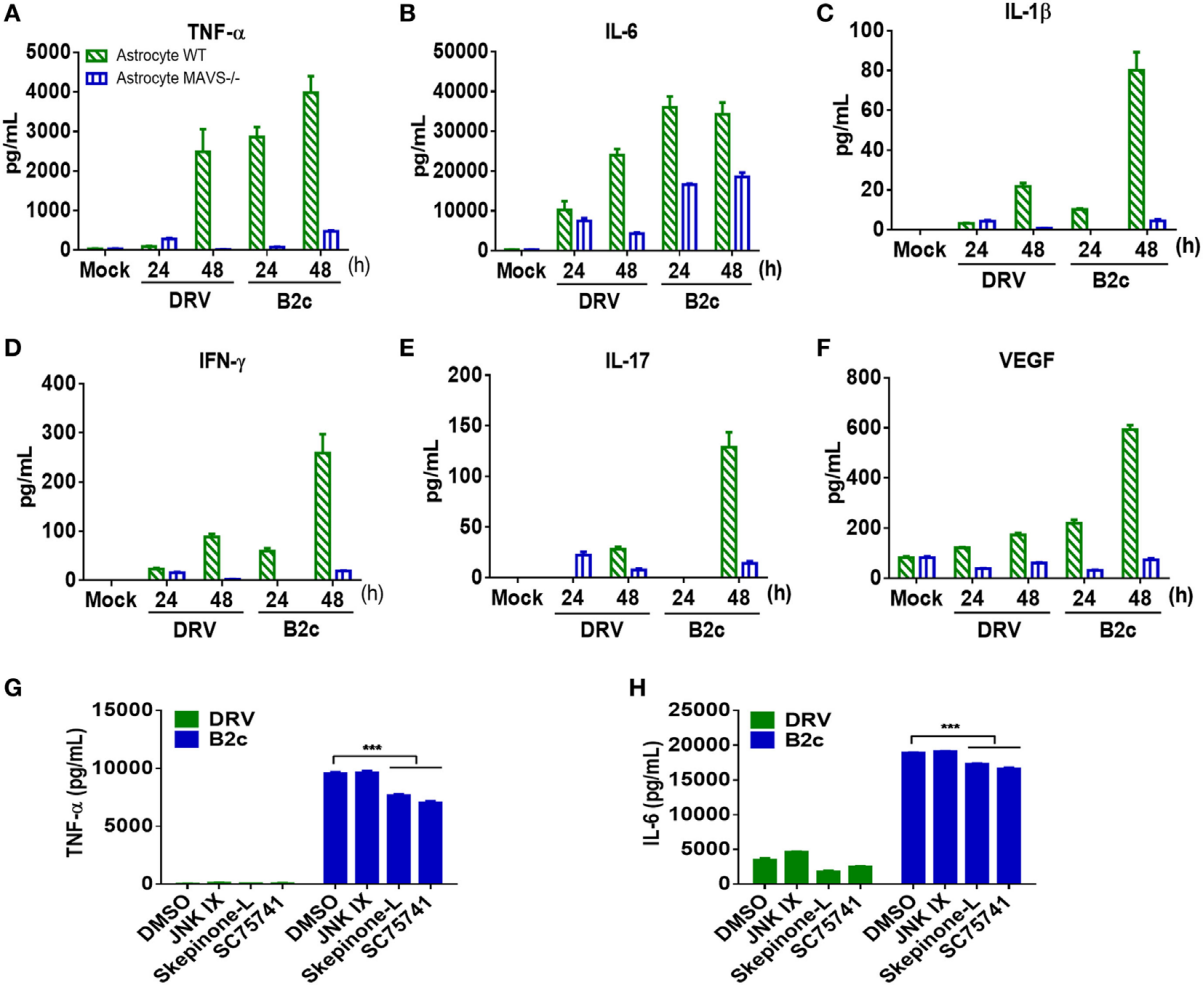
Cytokine expression in astrocytes infected by lab-attenuated rabies virus (RABV) is dependent on the mitochondrial antiviral-signaling protein (MAVS)-p38/NF-κB pathway. **(A–F)** Wild-type (wt) or MAVS−/− astrocytes were infected with DRV or B2c at an MOI of 1. At the indicated time points, the cell supernatants were collected, and TNF-α, IL-6, IL-1β, interferon-γ, IL-17, and vascular endothelial growth factor levels were determined by protein assay. **(G,H)** Wt astrocytes were treated with JNK inhibitor IX (JNK inhibitor), Skepinone-L (p38 inhibitor), sc75741 (NF-κB inhibitor), or DMSO (mock), after being infected with DRV and B2c at an MOI of 1. At 48 h p.i., the concentrations of TNF-α and IL-6 in the cell supernatant were measured with ELISA kits.

Astrocytes are one of the major sources of inflammatory cytokines in the CNS post viral infection, and these cytokines play an important role in regulating BBB permeability. To investigate RABV-induced cytokine production, primary astrocytes from wt and MAVS−/− mice were prepared and infected with DRV or B2c at an MOI of 0.01. At indicated time points, the cell culture supernatants were harvested and analyzed with a cytokine array kit. The results showed that B2c induced significantly higher levels of cytokine expression, namely, TNF-α, IL-6, IL-1β, IFN-γ, IL-17, and VEGF expression, than DRV in wt and MAVS−/− astrocytes. The levels of cytokine expression in MAVS−/− astrocytes were significantly lower than those in wt astrocytes, indicating that RABV-induced inflammatory cytokine production in astrocytes is dependent on the MAVS signaling pathway (Figures 6A–F).

The specific pathway through which RABV induces cytokine production was subsequently identified in astrocytes. A previous study demonstrated that RABV induces cytokine production in macrophages mainly through p38, JNK, and NF-κB pathways (46, 47). Thus, primary astrocytes were treated with p38, JNK, and NF-κB pathway inhibitors Skepinone-L, JNK IX, and sc75741, respectively, and the concentrations of TNF-α and IL-6 in the cell supernatant were measured by ELISA. The results showed that Skepinone-L and sc75741 caused greater reductions in TNF-α (Figure 6G) and IL-6 (Figure 6H) protein levels than JNK IX in B2c-infected astrocytes. None of these inhibitors significantly altered the expression levels of TNF-α and IL-6 in DRV-infected astrocytes. These findings suggest that lab-attenuated RABV induces cytokine expression in astrocytes mainly through the p38 and NF-κB pathways and that wt RABV suppresses cytokine production in astrocytes.

### Cytokines Produced in Astrocytes Regulate BBB Permeability

**Figure 7:**
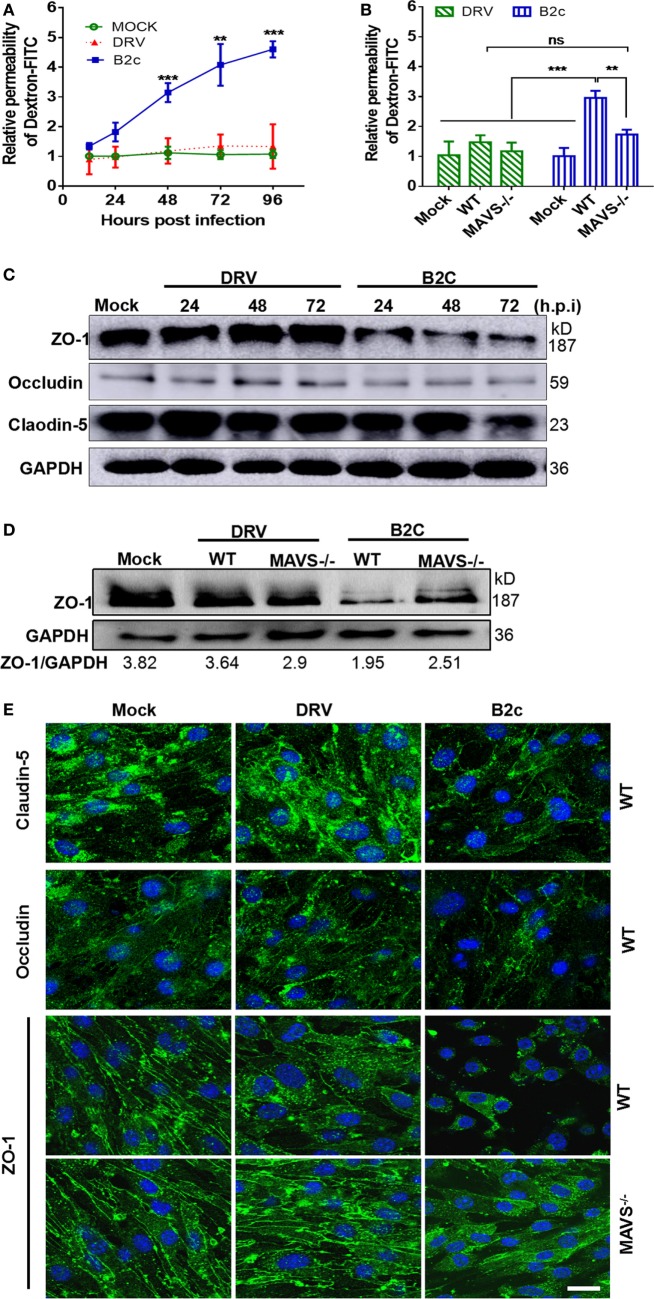
Inflammatory cytokines produced by astrocytes play a critical role in regulating blood–brain barrier permeability in rabies virus infection. Wild-type or mitochondrial antiviral-signaling protein (MAVS−/−) astrocytes were infected with DRV or B2c at an MOI of 1 or mock infected. **(A,B)** At the indicated time points, the cell supernatants were collected and UV-inactivated, then they were cocultured with b.END3 cell for 48 h. The cells were treated with Dextran-FITC at a dose of 0.1 µg/mL for 30 min, and then the Dextran-FITC fluorescence values in the media from the upper and lower chambers were determined by a fluorimeter. **(C,D)** The b.END3 cells were lysed with RIPA buffer, and the protein expression levels were determined by Western blot using antibodies against Zoluna occludens-1 (ZO-1), Claudin-5, Occludin, or GAPDH. **(E)** The b.END3 cells were fixed with PFA, stained with antibodies against Claudin-5, Occludin, ZO-1, or DAPI, and then observed under an immunofluorescence microscope. The scale bars represent 50 µm.

Our previous studies demonstrated that the chemokines/cytokines induced by RABV infection are responsible for reducing TJ protein expression and enhancing BBB permeability (39). To investigate the effect of these cytokines on BBB permeability, a mouse brain microvascular endothelial cell line b.END3, was cocultured with UV-inactivated supernatants infected with DRV or B2c and collected them at different time points after infection. Treatment with the supernatants from B2c-infected astrocytes for 48 h (Figure 7A) induced significant increase in Dextran-FITC infiltration from 48 to 96 h p.i. Notably, treatment with the supernatants from B2c-infected MAVS−/− astrocytes elicited significant increases in Dextran-FITC permeability at 48 h p.i. (Figure 7B). No significant increases in Dextran-FITC permeability were observed after treatment with the supernatants from wt or MAVS−/− astrocytes infected with DRV.

To gain an insight into the mechanisms by which lab-attenuated RABV enhances BBB permeability, the expression levels of the indicated TJ proteins (Claudin-5, Occludin, and ZO-1) were assessed in astrocytes by Western blot. It was found that the expression levels of Claudin-5 and Occludin were unchanged, while ZO-1 was significantly reduced after the cells were treated with the supernatants from B2c-infected wt astrocyte (Figure 7C). However, this reduction was attenuated in cells treated with the supernatants from MAVS−/− astrocytes infected with B2c. No significant changes in ZO-1 expression were observed in b.END3 cells treated with supernatants from wt or MAVS−/− astrocyte infected with DRV (Figure 7D).

Similarly, IFA results showed that ZO-1 expression was significantly decreased in b.END3 cells cocultured with the supernatants from B2c-infected astrocytes collected at 48 h p.i. (Figure 7E). ZO-1 fluorescence intensity was only slightly decreased in b.END3 cells cocultured with the supernatants from B2c-infected MAVS−/− astrocytes collected at 48 h p.i., indicating that cell-to-cell contact was not significantly decreased in these cells. Moreover, no ZO-1 degradation was observed in b.END3 cells cocultured with the supernatants from wt or MAVS−/− astrocytes infected with DRV. To be noted, no significant changes in Claudin-5 and Occludin expression were detected in either wt or MAVS−/− astrocytes upon RABV infection (Figures 7C,E). Together, these results illustrate that lab-attenuated, but not wt, RABV upregulates the production of inflammatory cytokines in astrocytes, resulting in ZO-1 degradation in BMECs and subsequent increases in BBB permeability. Furthermore, these results indicate that cytokine production is dependent on MAVS signaling pathway.

## Discussion

Astrocytes play critical roles in host defense during viral infections of the CNS (7). PRR activation in astrocytes results in the expression of many immune mediators, including type I IFNs and inflammatory cytokines (6, 7). During infections caused by pathogens for which glia are not susceptible targets, activation of the innate immune system caused by pathogen recognition in astrocytes may promote antiviral immune responses in susceptible neurons, as well as CNS leukocyte trafficking (3, 5, 7). In an early report, primary murine, feline, and human astrocytes were infected with wt (SRV) and lab-attenuated RABV (ERA) (34), after which viral loads and replication were assessed by infectivity assay and immunofluorescence. The results showed that astrocytes can be infected by RABV, suggesting that astrocytes may play a role in viral spread and persistence and/or neuronal dysfunction (34). However, in that study, viral loads and replication were evaluated only at the early time point after infection (3 d.p.i.). In this present study, the growth of DRV with that of B2c, which were used as a pair of wt and lab-attenuated RABV in previous studies (38–40), was compared in a long-term experiment. Surprisingly, we found that lab-attenuated RABV, but not wt RABV, caused abortive replication in astrocytes, a feature that may be associated with the ability of the virus to evade the innate immune response.

Productive infection of the astrocyte is critical for neurotropic pathogens to induce encephalitis. Astrocytes sensed viral entry into the CNS and mounted a type I IFN response, which rapidly restricts the virus after neuronal transport into the CNS. Previous studies demonstrated that TLR3−/− astrocytes were more permissive to HSV infection and caused severe symptom of encephalitis and tissue damage, which was due to impaired type I IFN production in the absence of TLR3 (30). A recently work found that abortively infected astrocytes are the major producers of IFN-β after infection of the brain with diverse neurotropic viruses, including TMEV, RABV, and vesicular stomatitis virus (VSV) (35). Consistent with these studies, we also found that the abortive infection of lab-attenuated RABV in astrocytes was related to its ability to activate IFN signaling pathway. Basal ISG expression levels are an important determinant of susceptibility to viral infection. We have found that astrocytes have higher basal expression levels of the mRNAs encoding ISG proteins, such MDA5 and STAT1, and other molecules crucial for recognizing viral invasion and creating an more antiviral environment than neurons (9), which may explain why lab-attenuated RABV activates the innate immune response to a degree sufficient to restrict viral replication in astrocytes but not in neurons.

Double-stranded RNA is a viral product that plays an essential role in inducing innate immunity, which leads to the production of type I IFNs and the activation of hundreds of ISGs. Early biochemical studies of viral replication suggested that most viruses produce dsRNAs (13). However, in 2006, Weber et al. reported that dsRNA could be detected by IFA in cells infected with positive-stranded RNA viruses, but not with negative-stranded RNA viruses (48). This notion was challenged by two other studies on dsRNA production in cells infected with negative-stranded RNA viruses (13, 48). Moreover, a recent study demonstrated the dsRNA formation in cells infected with several negative-stranded RNA viruses, such as VSV, measles virus (MeV), and influenza A virus, although the intensity of the staining of dsRNA tended to be weaker in cells infected with negative-stranded RNA viruses when compared with those infected with positive-stranded RNA viruses (13). Consistent with this finding, the production of dsRNA was detected in both lab-attenuated and wt RABV-infected astrocytes, although lab-attenuated RABV produced significantly more dsRNA in the cytoplasm than wt RABV.

We attempted to investigate why lab-attenuated RABV produces more dsRNA than wt RABV. Pfaller et al. found that dsRNA expression was much lower in cells infected with wt MeV than in cells infected with a mutant MeV whose C protein was knocked out which is known to control genome replication and transcription (49). Similarly, Takeuchi et al. observed dsRNA formation in cells infected with Sendai virus (50) with C protein knocked out but not in cells infected with wt SeV, indicating that the C protein limits or masks dsRNA production (51). Both MeV and SeV are within the family *Paramyxoviridae*, thus it is possible that their C protein possess the similar function to subvert the production of dsRNA. Ebola virus protein VP35 adopts a unique strategy to mask key cellular recognition sites on dsRNA (52). A recently study showed that the coronavirus endonuclease (EndoU) activity is the key to prevent early induction of dsRNA. Replication of EndoU-deficient coronaviruses is greatly attenuated *in vivo* and severely restricted in primary cells even during the early phase of virus infection (53). In this present study, by exchanging viral genes between wt and lab-attenuated RABV, we found that single N, P, and G of wt RABV could not suppress dsRNA formation (data not shown). However, multiple viral proteins of RABV may work together to limit the production of dsRNA. Further studies are needed to elucidate the mechanism through which wt RABV restricts dsRNA formation and thus evades recognition by the innate immune system in infected cells.

The BBB, which is composed of specialized BMECs joined by TJs and ensheathed by astrocytes and pericytes, plays an important role in protecting the CNS. Our previous studies have shown that RABV does not infect BMECs, nor does it modulate TJ protein expression in BMECs (39). However, brain extracts prepared from mice infected with lab-attenuated RABV but not wt RABV reduced TJ protein expression in BMECs, indicating that the above enhancements of BBB permeability and reductions in TJ protein expression are not caused by RABV infection. Rather, they are caused by virus-induced inflammatory chemokines/cytokines. The innate immune mechanisms that regulate BBB function in the setting of infectious diseases have been appreciated only recently. Multiple inflammatory cytokines, including TNF-α, IL-6, IL-1β, IFN-γ, IL-17, and VEGF, disrupt BBB and TJ integrity in BMECs (46, 50, 54–56), and inflammatory cytokine signaling at the BBB during infection facilitates leukocyte trafficking into the CNS, which is essential for the clearance of many pathogens (39, 41). Our present study demonstrates that lab-attenuated RABV induces production of several inflammatory cytokines in astrocytes, especially TNF-α, IL-6, IL-1β, IFN-γ, IL-17, and VEGF, which cause disruption of the BBB and TJ integrity. Our findings suggest that astrocytes play a critical role in regulating BBB permeability as a major source of cytokines during viral infection. Furthermore, we found that the production of inflammatory cytokines in astrocytes by lab-attenuated RABV was dependent on MAVS signaling pathway, underscoring the critical role of MAVS signaling in defensing against RABV infection in CNS.

## Conclusion

**Figure 8:**
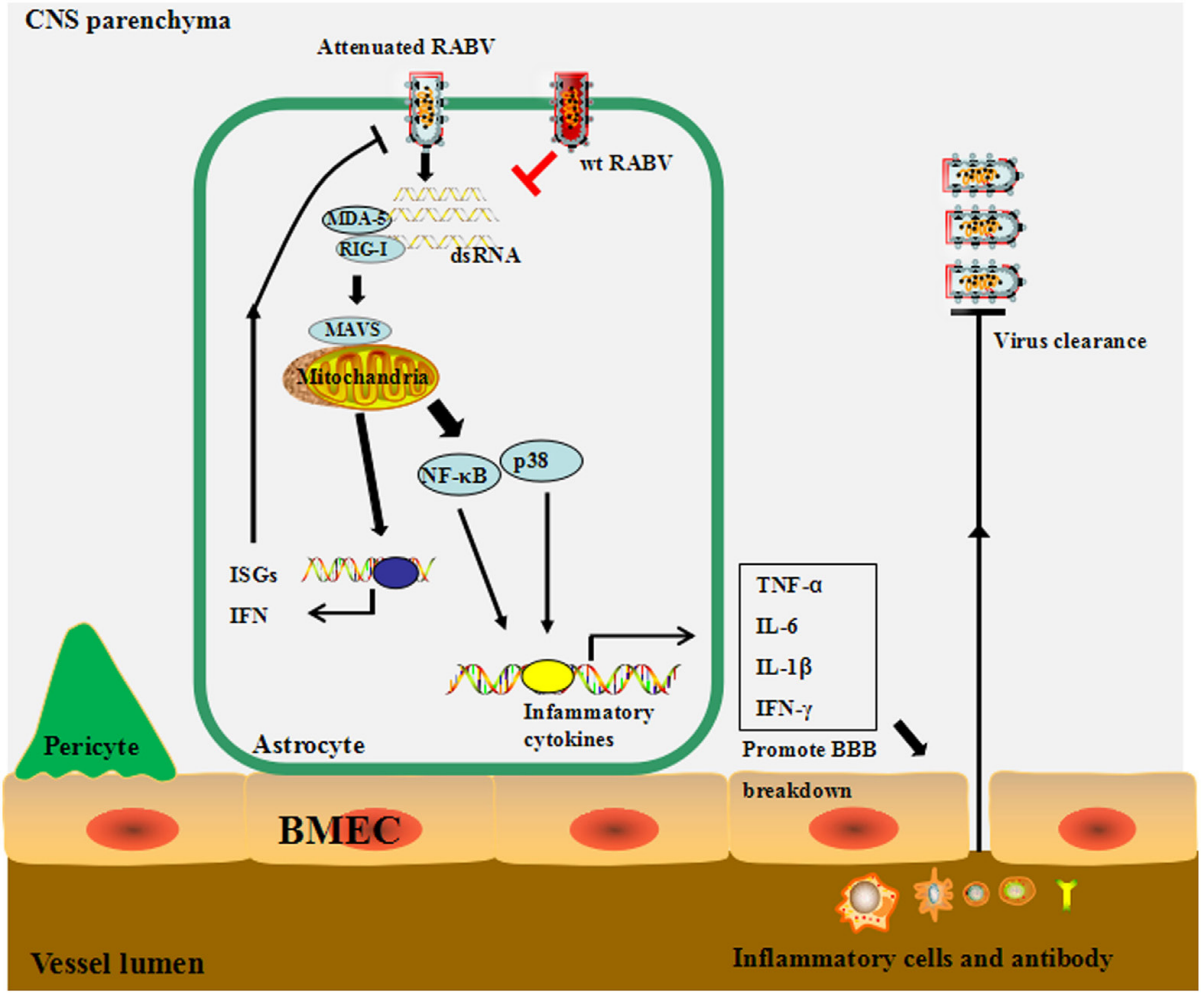
The proposed model for the mechanism through which wild-type (wt) and lab-attenuated rabies virus (RABV) infect astrocytes. During infection, lab-attenuated RABV produces double-stranded RNA (dsRNA), which is recognized by retinoic acid-inducible gene I (RIG-I)/melanoma differentiation-associated protein 5 (MDA5). Activation of mitochondrial antiviral-signaling protein (MAVS), the common adaptor protein for RIG-I and MDA5, leads to enhanced production of interferon, as well as some IFN-stimulated genes, which limit RABV replication and spread. MAVS activation stimulates the p38 and NF-κB signaling pathways and induces cytokine production in astrocytes. Inflammatory cytokines promote blood–brain barrier (BBB) permeability, enabling peripheral inflammatory cells and antibodies to infiltrate into the central nervous system, thereby facilitating RABV clearance. In contrast, wt RABV prevents activation of the MAVS signaling pathway by restricting dsRNA production.

Based on the results, we propose the following model: lab-attenuated RABV produces dsRNA recognized by RIG-I, MDA5, or both, resulting in the activation of the MAVS signaling pathway in astrocytes. IFN expression induces the transcription of hundreds of ISGs to inhibit RABV replication in astrocytes and causes the abortive infection by lab-attenuated RABV. The inflammatory cytokines induced by lab-attenuated RABV enhance BBB permeability, enabling immune cells and antibodies to infiltrate the CNS and facilitate RABV clearance. Conversely, wt RABV restricts dsRNA production and then evades recognition by the innate immune system, resulting in persistent viral replication in astrocytes (Figure 8).

## Ethics Statement

All experiments involving mice were performed in accordance with the recommendations in the Guide for the Care and Use of Laboratory Animals of the Ministry of Science and Technology of China and were approved by the Scientific Ethics Committee of Huazhong Agricultural University (permit number HZAUMO-2015-019).

## Author Contributions

Conceived and designed the experiments: BT and LZ. Performed the experiments: MZ, YY, LY, ZL, DT, and KW. Analyzed the data: BT, MC, HC, ZF, and LZ. Wrote the article: BT, MZ, ZF, and LZ.

## Conflict of Interest Statement

The authors declare that the research was conducted in the absence of any commercial or financial relationships that could be as a potential conflict of interest.
